# Learning to Expect: Predicting Sounds During Movement Is Related to Sensorimotor Association During Listening

**DOI:** 10.3389/fnhum.2019.00215

**Published:** 2019-07-04

**Authors:** Jed D. Burgess, Brendan P. Major, Claire McNeel, Gillian M. Clark, Jarrad A. G. Lum, Peter G. Enticott

**Affiliations:** Cognitive Neuroscience Unit, School of Psychology, Deakin University, Geelong, VIC, Australia

**Keywords:** sensory prediction, sensorimotor association, predictive comparison, TMS, EEG

## Abstract

Sensory experiences, such as sound, often result from our motor actions. Over time, repeated sound-producing performance can generate sensorimotor associations. However, it is not clear how sensory and motor information are associated. Here, we explore if sensory prediction is associated with the formation of sensorimotor associations during a learning task. We recorded event-related potentials (ERPs) while participants produced index and little finger-swipes on a bespoke device, generating novel sounds. ERPs were also obtained as participants heard those sounds played back. Peak suppression was compared to assess sensory prediction. Additionally, transcranial magnetic stimulation (TMS) was used during listening to generate finger-motor evoked potentials (MEPs). MEPs were recorded before and after training upon hearing these sounds, and then compared to reveal sensorimotor associations. Finally, we explored the relationship between these components. Results demonstrated that an increased positive-going peak (e.g., P2) and a suppressed negative-going peak (e.g., N2) were recorded during action, revealing some sensory prediction outcomes (P2: *p* = 0.050, $\eta_{\text{p}}^{2}$ = 0.208; N2: *p* = 0.001, $\eta_{\text{p}}^{2}$ = 0.474). Increased MEPs were also observed upon hearing congruent sounds compared with incongruent sounds (i.e., associated to a finger), demonstrating precise sensorimotor associations that were not present before learning (Index finger: *p* < 0.001, $\eta_{\text{p}}^{2}$ = 0.614; Little finger: *p* < 0.001, $\eta_{\text{p}}^{2}$ = 0.529). Consistent with our broad hypotheses, a negative association between the MEPs in one finger during listening and ERPs during performance of the other was observed (Index finger MEPs and Fz N1 action ERPs; *r* = −0.655, *p* = 0.003). Overall, data suggest that predictive mechanisms are associated with the fine-tuning of sensorimotor associations.

## Introduction

Typically, sounds are produced by our motor actions. Through performance, the *cause and effect* relationship between motor and sound information can become evident. Over time, repeated experience can generate sensorimotor associations and the innervation of motor and sensory data. It is proposed that these sensorimotor associations assist with precise motor control (Shadmehr et al., 2010). For instance, when learning to press a key on the piano, a student will begin to recognize that sounds are aligned with keys, and these are (typically) activated by specific finger movements. Thus, should one desire to hear those sounds, the known finger movements should be executed.

In humans, evidence of sensorimotor association is usually demonstrated during sensory processing and activation of motor-brain regions. This can be achieved with transcranial magnetic stimulation (TMS) to the primary motor cortex (M1). TMS can be applied when listening to sounds that are associated with a particular action, such as clicking one’s fingers (for review, see Aglioti and Pazzaglia, 2010). Upon stimulation, hearing the sound activates the motor program that formerly produced it, and heightened M1 excitability is revealed. Subsequently, larger motor evoked potentials (MEPs) are measured at the peripheral muscle involved in the action when compared to a baseline condition. The response, where known sounds activate motor regions and specific corticospinal tracts, is called *auditory-motor resonance* (AMR; for review, see Burgess et al., 2017).

AMR has been demonstrated extensively. Hearing piano sounds increases MEPs in finger muscles of pianists when compared to non-pianists (Furukawa et al., 2017), highlighting the importance of experience. Similarly, MEPs in tongue muscles are facilitated in response to speech listening (D’Ausilio et al., 2014; Nuttall et al., 2017). Sensorimotor training has even produced novel MEP *disassociations* (Ticini et al., 2011, 2019). That is, an index or little finger button-press can be associated with distinct sounds during training. Afterwards, hearing the index-*congruent* sound generates larger MEPs in the index muscle than hearing the *incongruent* little-finger sound. Together, these disassociations highlight the precision of the AMR networks.

However, how sensory and motor systems integrate to produce a sensorimotor association is unclear. Associating a sound with a motor action must overcome inherent time delays that are met during sensory processing. For example, the action that generated *this* sound occurred in the past. Thus, in the first instance, there is a temporal disconnect between the motor and sensory aspects that might seem to work against the formation of an experience-dependent association. To overcome this issue (and others), it is suggested the central nervous system (CNS) predicts impending sensory changes during action (Shadmehr et al., 2010; Burgess et al., 2017).

Sensory predictions are critical for effective and fluid behavior (for review, see Sawtell, 2017; Schneider and Mooney, 2018; Straka et al., 2018). From a prediction perspective, smooth movement is achieved over time *via* a comparison between desired (predicted) and produced (actual) sensory consequences. When predicted and actual stimuli are compared, expected sensory consequences are attenuated or suppressed (Aliu et al., 2009; Kilteni and Ehrsson, 2017). Unexpected or novel stimuli, however, are not (Knolle et al., 2013; Mathias et al., 2015). In turn, the feedback generated by this process helps make the movement more efficient and accurate.

In humans, electroencephalography (EEG) and event-related potentials (ERPs) can be used to indicate the sensory prediction processes during action (for reviews, see Woodman, 2010; Bendixen et al., 2012; Joos et al., 2014; Horváth, 2015). When individuals produce sounds (e.g., speaking, arm, leg, or finger-press generated sounds), which are presumably highly predictable, the suppression of a negative-going peak around 100 ms is often demonstrated when compared to the same audition-obtained peak (Ford et al., 2007; Baess et al., 2011; Van Elk et al., 2014). This suppression represents attenuation of those expected sensory consequences during action. Alternatively, the accentuation of the ERP peak recorded during listening is thought to represent the absence of sensory prediction. For the CNS, it indicates that the incoming sounds are unexpected and important or might even be produced by someone else (Haggard, 2017).

Beyond the attenuation of the incoming sounds during a performance, as indexed by N1 peak suppression, modulation of other peaks during an action have also been discussed in terms of sensory prediction. While it is not well understood (Crowley and Colrain, 2004; Tong et al., 2009) and relatively unclear what sensory prediction outcomes they could represent (Horváth, 2015; Pinheiro et al., 2018), modulation of the positive-going P2 peak around 200 ms is also reported during action (Chen et al., 2012; Knolle et al., 2013; Timm et al., 2014; Ghio et al., 2018). Effects include decreased suppression for delayed stimulus onsets (Behroozmand et al., 2011; Pereira et al., 2014), pitch-shifted sounds (Behroozmand et al., 2014), or trained sounds (Reinke et al., 2003; Tong et al., 2009). Enhancement of an earlier P1 component (Boutonnet and Lupyan, 2015), and also suppression of latent N2 peaks (Knolle et al., 2013; Mathias et al., 2015) or related (Horváth et al., 2008) mismatch negativity (MMN) is also reported (for review, see Näätänen et al., 2007; Winkler, 2007; Garrido et al., 2009; Bartha-Doering et al., 2015). Altogether, changes in negative and positive-going ERP peaks across action and audition recordings are considered to reflect the sensory prediction mechanisms and their outcomes.

In sum, there is reliable evidence for sensory prediction and sensorimotor associations. However, few reports investigate how they interact. Outside of studies exploring the cerebellum’s role in sensory prediction during visually perturbed actions (Miall et al., 2007; Izawa et al., 2012; Yavari et al., 2016), very few studies show the interaction between auditory predictions and audio-motor associations following a learning task. Here, we explore for the first time, from an auditory perspective in humans, how motor behavior, sensory prediction markers, and sensorimotor associations are correlated within a single paradigm.

To investigate, an auditory-motor task was designed. This required participants to make an index or little finger-swipe movement on a bespoke device. Activation of one of two switches would result in playback of a sound *via* in-ear headphones. The sensory prediction mechanisms were assessed *via* ERPs, as demonstrated by changes in ERP suppression across action and audition stages. The sensorimotor associations, however, were assessed *via* TMS-induced MEPs during listening, before and after the training period. Finally, we explored the relationship between sensory prediction, sensorimotor association, and motor behavior [e.g., electromyography (EMG) recordings during swipes].

We hypothesize that sensory prediction will be evident. That is, increases in ERP suppression for a negative-going peak such as the N1 (e.g., Van Elk et al., 2014) or a later N2 peak are expected to be present during action (e.g., Mathias et al., 2015; i.e., given the novelty of the action and relatively long swipe duration, we investigated three broad ERP peak windows). Despite the conflicting evidence regarding a positive peak usually around 200 ms, we hypothesized that a decrease in suppression of the first positive-going peak (e.g., P2) will be observed during the performance, reflecting a signal to maintain perceptual gaps during sensory prediction processes (e.g., Wang et al., 2014). AMR was also expected to be revealed. Specifically, we hypothesized that congruent sounds will generate larger MEPs when compared with those recorded upon hearing incongruent sounds (e.g., Ticini et al., 2011). Finally, given the close ties between sensory prediction and sensorimotor association under theoretical accounts (Wolpert and Kawato, 1998; Burgess et al., 2017), we hypothesized that some correlations between the ERP and EMG data during action with MEPs during listening will be present (see Supplementary Figure S1 in Supplementary Materials for illustration of the experimental hypotheses).

## Materials and Methods

### Participants

We recruited 18 healthy adult participants, including eight females [mean age = 27.33 years (SD = 5.28)]. Participants did not reveal a history of neurological or psychiatric illnesses (i.e., self-reported). All participants indicated normal hearing and reported right-handedness, as confirmed by the Edinburgh handedness inventory (Oldfield, 1971). Participants were also screened to ensure they met TMS safety standards (Rossi et al., 2009, 2011). Participants provided informed written consent in accordance with the Declaration of Helsinki. Participants were compensated for their time. The research was approved by the Deakin University Human Research Ethics Committee (2015-034).

### Experimental Design and Procedure

**Figure 1:**
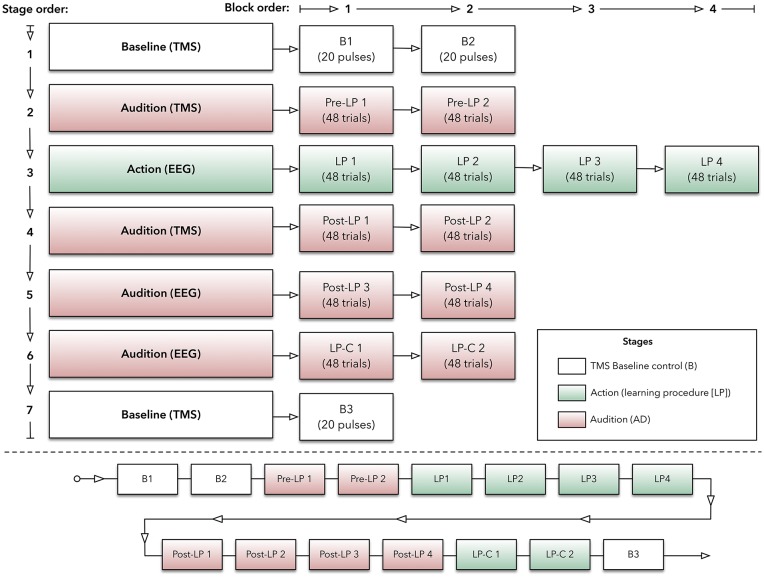
Experimental protocol. Top panel: outlined here are the experimental stages, which are made up with blocks of transcranial magnetic stimulation (TMS) or electroencephalography (EEG) trials. Bottom panel: this panel indicates the order of blocks (within their respective stage) across the experiment.

The experimental paradigm was based on an ERP investigation (Ford et al., 2010) and a TMS motor-learning study (Ticini et al., 2011). It consisted of two main stages: (1) *Action* and (2) *Audition*. During the action stage, participants produced sounds by making finger-swipes on the experimental device, while EEG and EMG techniques recorded CNS activity. The audition stage, however, used TMS and EMG to record CNS activity when participants passively listened to sounds that were played back *via* the device. These stages were also comprised of individual blocks to help minimize issues with participant attention waning (e.g., Finkbeiner et al., 2016). Overall, participants produced sounds (i.e., action) or heard them (i.e., audition) while EEG, EMG, and TMS recorded CNS activity within separate experimental blocks (see Figure 1 for illustration of the protocol).

**Figure 2:**
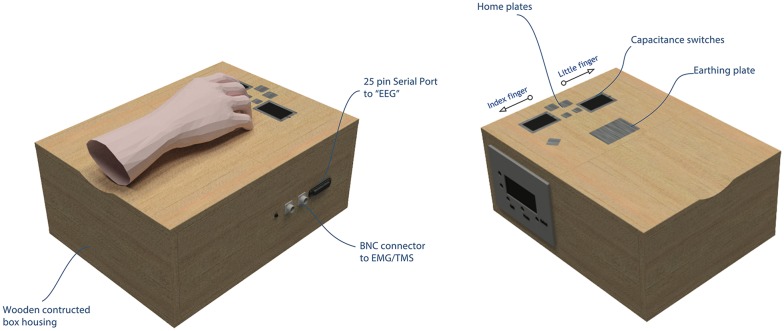
The AMRJ. Left panel: during the action stage, the custom-made AMRJ device (abbreviation used as the proper name) uses finger movement to activate the respective capacitance switch. This activation produces playback of an original sound *via* the in-ear headphones (i.e., there are two sounds). During the audition stage, the AMRJ can produce a pseudorandom playback of the sounds without the need for finger switch activation. In either mode, the AMRJ triggers the respective recording devices *via* the serial port or BNC connectors. Right panel: swipes are produced by either the (right-hand) index or little fingers. For the index finger, a swipe is produced upon the touch of the left capacitance switch, from inside-to-outside of the switch (e.g., left direction). For the little finger, a right directed finger-swipe initiates the respective switch (i.e., over the right-hand side switch from inside-to-outside).

Participants sat comfortably in a chair. The custom-made device, labeled the AMRJ (outlined below and illustrated in Figure 2), was placed in front of them. After each neuroscientific technique setup was completed (described below), the experiment began with the *Baseline* stage. This stage consisted of two blocks (*B1* and *B2*). These blocks and the final Baseline block (*B3*) were designed to measure transient-state influences (Schmidt et al., 2009) and potential cumulative effects of single-pulse TMS (Pellicciari et al., 2016). Each block had 20 TMS pulses with a 4-s inter-stimulus interval and 5-s inter-block interval. Each block lasted approximately 2 min.

Following a 1-min break, the *Pre-learning* stage (*Pre-LP*) began. The Pre-LP stage was comprised of two blocks (so-called *Pre-LP 1* and *Pre-LP 2*). Each block established a baseline of MEPs and, therefore, AMR. Participants listened to a quasi-randomized block of 48 sound samples (i.e., 48 trials in a block; sounds are described below). During listening, TMS was applied to M1, and MEPs were recorded from both the index and little finger muscles simultaneously. Each block lasted approximately 5 min, and a 1-min break between blocks was provided.

**Figure 3:**
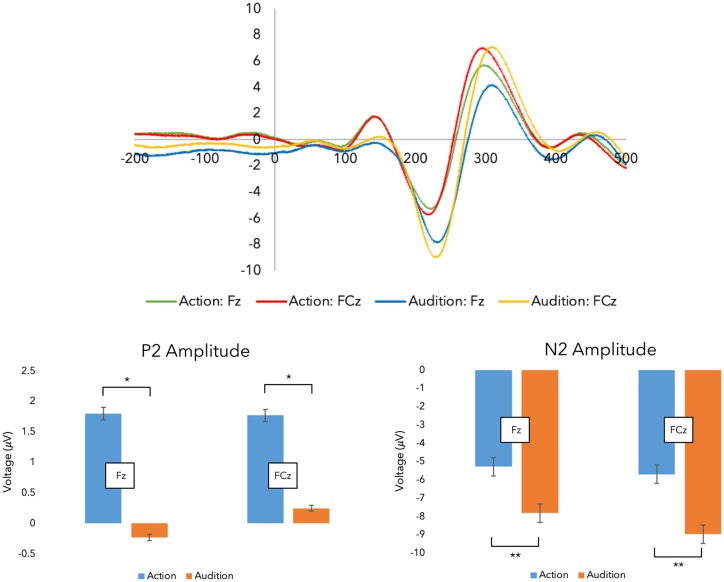
Grand average event-related potentials (ERPs). Top panel: grand average ERPs were recorded during the experimental stages across both fingers.Shown here are the traces for the Fz and FCz electrodes (i.e., the action stage data has been corrected for movement-related potentials, and both finger action-sounds have been combined to illustrate the ERPs). Bottom panel: here, the peak amplitude within each time window are plotted (*p < 0.05, **p < 0.001; error bars represent standard error of the mean).

After another 1-min break, the *Learning Procedure* (LP) stage began. This stage was intended to associate finger-swipes and sounds, and participants produced the sounds *via* finger-swipes. There were four LP blocks (*LP 1*, *LP 2*, *LP 3*, and *LP 4*). Each block required participants to perform 48 swipe movements with their index or little fingers across the corresponding switch, which would generate the sounds. Beginning with the inside switch-edge, each finger would move towards the outer edge of the device (see Figure 3 for illustration). The index finger-swipe was toward the left-hand side of the device, while the little finger generated a swipe towards the right. The starting position required the middle and ring fingers to be positioned over the two *home* plates. This allowed the index and little fingers to be aligned with the inside edge of the respective switch. Before testing, the experimenter provided an example of both finger-swipes. Each swipe needed to be at least 300 ms in duration for sound playback to occur. Participants were instructed to modulate their speed to ensure swipe time was a minimum of 300 ms.

Once sound playback had finished after each swipe, participants returned to the original starting position. At the beginning of each LP block, participants were instructed to begin another swipe only after a self-timed 3-s break had expired. This break was designed to assuage the concern of fatigue during sensorimotor learning tasks (Bock et al., 2005; McDonnell and Ridding, 2006). If the experimenter observed that the participant began a swipe before the 3-s break had expired, participants were notified *via* a shoulder tap before the next swipe. This indicated they were to increase the rest period between trials.

To help promote motor learning, participants voluntarily chose which movement to execute (Herwig et al., 2007). Participants were asked to perform an approximately equivalent number of index and little finger-swipes (24 each) to mitigate any potential learning biases. This was observed, and participants were made aware of the swipe distribution-count between LP blocks. During testing, 50.51% of swipes were generated with the index finger while 49.49% were produced using the little finger. Within an LP block, the minimum index swipe count was 18, and for the little finger at least 20 swipes were produced. Each block lasted approximately 5-min, and a 1-min break between each LP block was used.

Following a 4-min break, the next stage was the post-learning stage named *Post-LP*. Like the Pre-LP blocks, these stages required listening to the 48 sound-trials over separate blocks (*Post-LP 1*, *Post-LP 2*, *Post-LP 3*, and *Post-LP 4*). Post-LP 1 and 2 blocks used TMS, while Post-LP 3 and 4 used EEG separately. TMS and EEG were used independently to minimize interference across recordings. A 1-min break between all blocks was added.

Participants then completed the *Learning Procedure-control* (*LP-C*) stage. Here, two LP-C blocks (*LP-C 1* and *LP-C 2*) were used to isolate the motor component within the ERP trace. Convention suggests that data associated with movement should be subtracted from the LP block ERPs. This is thought to improve the comparison with listening-derived ERPs (Martikainen et al., 2005; Ford et al., 2007, 2010, 2014; Baess et al., 2011; Van Elk et al., 2014). Therefore, the LP-C blocks used the same overall design as the LP but did not produce any sound following a swipe action. That is, swipe movements were produced; however, no sounds were played. During testing, 50.77% of swipes were generated with the index finger while 49.23% were produced using the little finger.

Lastly, another *Baseline* stage was completed (block *B3*). Like the initial Baseline blocks, four sets of five TMS pulses at the motor threshold (MT) were applied while MEPs were recorded.

Participants were asked to observe their right hand throughout the experiment to ensure a degree of uniformity across experimental stages. Throughout all listening blocks, participants were asked to pay attention to the sounds and indicate if they heard a control sound after sound playback has finished (i.e., no control sound was used during listening trials, however). In total, the experiment lasted approximately 90–120 min.

### The AMRJ

Manufactured by SPLat Controls (SPLat Controls, Seaford, VIC, Australia) and Maximum Design (Max Designs, Croydon North, VIC, Australia), the AMRJ consists of the MS121USB216 controller and MP3 Trigger printed circuit board. Intended for our experimental protocol, the AMRJ uses finger movement to activate inbuilt switches. There are two switches; one for the index finger and another for the little finger. These detect changes in electrical capacitance upon touch. They are capacitance switches and, therefore, do not require a force to activate. Mechanical button presses can produce unwanted sounds and even accentuate tactile information, which can affect ERP recordings (Horváth, 2014). The use of capacitance switches reduces the potential confound of tactile and other sound feedback on ERP recordings.

The device can also bypass switch activation and play a quasi-randomized sequence of the audio samples. In either mode, the AMRJ triggers EEG, EMG, and TMS equipment to help record data when needs require (see Figure 2 for illustration of the AMRJ, while more details regarding the switches and trigger design can be found in the Supplementary Materials).

### Sounds

Swipe movements were followed by one (of two) complex sounds at a stimulus onset asynchrony (SOA) of 10 ms. This delay would ensure all techniques were triggered simultaneously (where required). The sounds were recorded on Ableton 9.0 software (Ableton AG, Mitte, BER, Germany) using a Roland Juno 60 synthesizer (Roland Corporation, Hamamatsu, 22, Japan) at Otologic Studios (Toorak, VIC, Australia). One sound consisted of an approximate 500 Hz fundamental tone, as well as 250 Hz and 1,000 Hz overtones. This sound was heard as a *low* sound. The other sound, so-heard as the high sound, was comprised of an approximate 1,800 Hz fundamental tone, as well as 2,100 Hz and 4,000 Hz overtones (for sound spectrograms, see Supplementary Figure S2 in Supplementary Materials).

Assignment of sounds (low or high) to each switch (index or little finger) were counterbalanced across participants (Ticini et al., 2011). For example, some participants produced the high sound by an index finger-swipe, while others produced the low sound (by that finger-swipe). For statistical purposes, however, we refer to the MEPs recorded in the index finger as the congruent sound or sound associated with the index finger as the Congruent_FDI_ data. Since these sounds were pinned for each participant, the Congruent_FDI_ sound then becomes the incongruent sound for the little finger. Therefore, the Incongruent_ADM_ term refers to the MEPs obtained in the little finger during playback of the index finger sound. Similarly, the Congruent_ADM_ sound describes the MEPs obtained during playback of the little finger-congruent sound. Therefore, this sound becomes the index finger incongruent sound, and reflects the MEPs recorded in the index finger during the little finger-swipe sound playback (i.e., Incongruent_FDI_).

Sounds were played through Etymotic ER3-10 ABR insert earphones (Compumedics USA, Charlotte, NC, USA), *via* the AMRJ 3.5 mm stereo port, and amplified to 75 dB or slightly lower if individual comfort levels were exceeded (i.e., sound levels were determined *via* the inbuilt MP3 Trigger printed circuit board).

### EEG Setup and Data Extraction

During the EEG blocks, data were recorded using 12 Ag-AgCl sintered electrodes. EEG electrode sites comprised Fz, FCz, Pz, P3, and P4. Electrodes were also placed on both mastoids, and a ground electrode was placed on the forehead for off-line referencing. Both vertical and horizontal electrooculograms (EOGs) were recorded using electrodes above and below the left eye, and on the outer canthus of each eye. EEG data were obtained *via* a SynAmps RT system (Compumedics USA, Charlotte, NC, USA) and Curry 7.07xs (Compumedics USA, Charlotte, NC, USA). Data were sampled at 10 kHz, and impedance was kept below 5 kΩ for each electrode.

EEG and EOG data were analyzed offline in Curry 7.07xs (Compumedics USA, Charlotte, NC, USA). A 50 Hz notch filter and a band-pass filter between 0.5 and 15 Hz was applied. Data were re-referenced to the mean combination of the left and right mastoids. To minimize the influence of eye blinks on the ERP, horizontal and vertical EOG data were corrected using Curry’s covariate analysis tool.

Bad EEG periods exceeding ±75 μV in amplitude were detected. The 700 ms epochs around these (i.e., 200 ms prior and 500 ms after trigger) were removed from further analysis to obtain conservative EEG epochs. Epochs that exceeded a signal-to-noise ratio below 0.5 and above 2.5 in a 700 ms window time-locked to triggers were also excluded from further analysis. From a possible 7114 blocks, 21.9% or 1,556 were removed.

For each participant, epochs were labeled for Sound (Congruent_FDI_ or Congruent_ADM_), Block [(action or audition) LP 1, LP 2, LP 3, LP 4, Post-LP 3, Post-LP 4], and LP-C blocks (LP-C 1 and LP-C 2). Subsequent analyses of the epochs investigated the peak amplitude of the N1, P2, and N2 ERP components. These were respectively examined within an epoch of 50–150 ms (N1), 100–200 ms (P2), and 151–250 ms (N2) for each participant across each ERP recording.

### TMS Setup and Data Extraction

During TMS blocks, focal TMS pulses were delivered to the scalp over the left M1. A 70 mm figure-of-eight stimulation coil was used (Magstim Company, Whitland, UK), and the coil was connected to a Magstim 200 stimulator (Magstim Company, Whitland, UK). Using self-adhesive Ag-AgCl electrodes, TMS-induced MEPs were recorded from first dorsal interosseous (FDI) muscle and abductor minimi digiti muscle (ADM) muscles simultaneously. A ground electrode was placed on the dorsal surface of the wrist (i.e., ulna bone). The EMG signal was amplified by a PowerLab/4SP (ADInstruments, Colorado Springs, CO, USA), and data were sampled *via* a FE135 Dual Bio-amp (ADInstruments, Colorado Springs, CO, USA). A band-pass filter between 0.3 and 1,000 Hz was applied, as well as a mains 50 Hz notch filter.

The site on the scalp that produced the largest median (peak-to-peak) MEP in five consecutive trials from the right-FDI while at rest was defined as M1. Stimulation to M1 determined the MT. MT was defined here as the stimulation intensity that evoked a median peak-to-peak MEP of approximately 1 mV. The acceptable range was between 0.8 and 1.3 mV. Once a suitable stimulator output had been determined (e.g., indicating MEPs from M1 were within the 0.8–1.3 mV range), recordings were obtained from 10 trials using the FDI muscle while the hand was at rest. If the median MT MEP was not found to fall within the accepted range across those 10 trials, the MT process began again. The stimulator output ranged from 33% to 60% [*M* = 46% (*SD* = 7.6%)] of the maximum stimulator output. We also used the Baseline blocks (B1, B2, and B3) to ensure no significant changes in background corticospinal excitability occurred. Corticospinal excitability across the paradigm was stable as measured *via* a mean of TMS Baseline blocks (see Supplementary Materials for test details).

Due to the uniqueness and duration of swipe movements, we opted for a broad range of trigger-points to obtain MEPs during listening. We were unsure where peak AMR would be recorded during listening. It was foreseeable the sensorimotor association might involve an internal mapping that encodes the sound with either movement commencement, movement transition, swipe termination, or a variation of each (for related discussion, see Horváth, 2013). We could not, however, determine swipe time precisely, which might help calculate a suitable AMR time-window or trigger point for participants. For example, an individual might produce slower swipes than another, which suggests a longer TMS trigger latency is appropriate to assess the sensorimotor association. Thus, a variety of *static* TMS-trigger time points were used to overcome this issue.

Focal-TMS pulses were delivered to M1 at 50, 150, 300, or 450 ms from SOA in each of the 48 trials (i.e., 12 pulses at each 50, 150, 300, or 450 ms from SOA). While these triggers do not consider individual variability, we considered the AMR time-window suitable for swipes 300+ ms in duration. Additionally, we considered the time points helpful in exploring some basics regarding timing during the association process. Potentially, that is, how the brain overcomes sensory delays while integrating a present sound with a past action (for discussion on this point, see Hanuschkin et al., 2013; Giret et al., 2014; Keysers and Gazzola, 2014; Burgess et al., 2017).

As is recommended for MEP data (Schmidt et al., 2009), individual median peak-to-peak MEP amplitudes (mV) were extracted for each TMS block (Pre-LP 1, Pre-LP 2, Post-LP 1, Post-LP 2, B1, B2, and B3) across Muscle (FDI or ADM), Sound (Congruent_FDI_ or Congruent_ADM_), and Time point (50, 150, 300, and 450 ms). Approximately 252 MEPs were obtained for each participant. Missing data points in baseline blocks, due to faulty TMS-based triggers, were replaced by the median MEPs of the remaining blocks for those participants (this required the alteration of two data points or 3.7% of Baseline MEPs collected across all participants). Also, one participant’s Post-LP 1 block was corrupt, requiring the (presumably comparable) Post-LP 2 dataset to be used (i.e., alteration of 1.4% of total MEPs averaged across all participants).

To minimize the influence on tests of normality, extreme outliers at a sample level were reduced to one value above the next highest data point (Tabachnick and Fidell, 2006). From the FDI muscle, 1.2% of MEP recordings were altered, while 1.9% of the ADM raw data. Tests of normality, histograms, as well as stem and leaf plots, were inspected and were considered satisfactory for parametric data analyses.

### Statistical Analyses

#### Testing Sensory Prediction

To assess if sensory prediction mechanisms are present, we investigated changes in N1, P2, and N2 peaks across action and audition stages. We examined each peak component separately *via* 2 (Finger: index or little) × 2 (Stage: Action or Audition) × 2 (Electrode: Fz or FCz) ANOVAs. A main effect for Stage will reveal sensory prediction mechanisms, with *post hoc* tests showing that suppression of ERP peaks is modulated by hearing the sounds across action and audition stages.

#### Testing Sensorimotor Associations

To assess the development of AMR, a 2 (Finger: Index or Little) × 4 (Audition block: Pre-LP 1–2 or Post-LP 1–2) × 4 (Time point: 50, 150, 300, or 450) × 2 (Sound: Congruent_FDI/ADM_ or Incongruent_FDI/ADM_) repeated-measures ANOVA was conducted on the normalized median peak-to-peak amplitude MEPs. This analysis compares Pre and Post-LP MEPs, which are recorded upon hearing congruent and incongruent sounds at a variety of time points. As stated, we were unsure where the largest AMR recordings would be revealed. Therefore, to show AMR, we expect the four-way ANOVA to reveal Audition block × Sound × Time point interactions. Subsequent *post hoc* tests should indicate larger MEPs in the Post-LP 1 and 2 blocks when the congruent sounds are heard at some time point in comparison to the incongruent sound MEPs at that time point. This effect, though, should not be present with the Pre-LP blocks (e.g., it is a *trained* effect).

#### Testing Sensory Prediction and Sensorimotor Association

Finally, we were interested in exploring the relationship between sensory prediction, sensorimotor association, behavioral data. Therefore, we used a Spearman’s correlation to investigate the association between: (a) EMG data obtained during the LP blocks with; (b) N1; (c) P2; (d) N2 ERP components also recorded during the LP training; (e) MEPs from post-LP blocks (i.e., we decided to omit pre-learning AMR data for the sake of clarity); (f) N1; (g) P2; and (h) N2 ERP components recorded during the audition stage. Here, we expect some correlations to exist between ERP and EMG data during action with MEPs during listening. This would indicate close ties between sensory prediction and sensorimotor association mechanisms.

All statistical analyses were carried out using SPSS 24 (IBM Corporation, Armonk, NY, USA) and analyses used a criterion of *p* < 0.05. All significant effects were investigated *via* follow-up ANOVA and pairwise comparisons (PCs) using a Bonferroni adjustment for multiple comparisons. Partial eta-squared effect sizes ($\eta_{\text{p}}^{2}$) were calculated to estimate the magnitude of an effect. Finally, in an effort towards conciseness, reporting of statistics is limited. For all test results, see Supplementary Materials.

## Results

### Testing Sensory Prediction

First, we investigated changes in sensory prediction. Figure 3 displays the grand-average ERP traces from SOA. Regarding the N1 peak, a three-way interaction between Finger, Stage, and Electrode was revealed (*F*_(1,17)_ = 6.031, *p* = 0.025, $\eta_{\text{p}}^{2}$ = 0.262) requiring further investigation (see Supplementary Materials). However, no main effects were present, suggesting action and audition peaks did not differ within this time window (50–150 ms).

Regarding the P2 peak, ANOVA revealed a main effect for Stage as hypothesized (*F*_(1,17)_ = 4.471, *p* = 0.050, $\eta_{\text{p}}^{2}$ = 0.208). *Post hoc* PCs, which were Bonferroni corrected, indicated that the action stage produced larger P2 peaks (*M* = 2.704, *SE* = 0.653) than the audition stage (*M* = 1.438, *SE* = 0.486). This suggests that some sensory prediction outcomes during action are being reflected in this peak.

As hypothesized, a main effect for Stage was demonstrated with the N2 peak data (*F*_(1,17)_ = 15.296, *p* = 0.001, $\eta_{\text{p}}^{2}$ = 0.474). PCs (Bonferroni corrected) revealed audition generated larger (more negative) peaks (*M* = −9.354*, SE* = 0.738) in comparison to action (*M* = −6.676, *SE* = 0.435). This suggests that a predictive process is being undertaken during action which suppresses the incoming sounds.

Altogether, we found evidence for some sensory prediction outcomes during finger-swipe movements.

### Testing Sensorimotor Association

Next, we determined if AMR developed (i.e., sensorimotor associations). Comparisons between Pre and Post-LP MEPs, which were recorded upon hearing congruent and incongruent sounds using the static time points, were explored.

The four-way ANOVA revealed a main effect for Time point (*F*_(3,51)_ = 4.809, *p* = 0.005, $\eta_{\text{p}}^{2}$ = 0.220). PCs (Bonferroni corrected) indicated the MEPs at the 50 ms time point (*M* = 0.681, *SE* = 0.060) were significantly larger than the 150 ms time point (*M* = 0.612, *SE* = 0.051; *p* = 0.003). This suggests motor-brain activity is high during the early stages of swipe-sound listening.

ANOVA also revealed a main effect for Audition blocks (*F*_(3,51)_ = 3.799, *p* = 0.016, $\eta_{\text{p}}^{2}$ = 0.183). Although PCs did not survive Bonferroni corrections, estimated means indicated that Post-LP 1 MEPs were, unexpectedly, the smallest recorded [Pre-LP 1: *M* = 0.708 (*SE* = 0.076); Pre-LP 2: *M* = 0.704 (*SE* = 0.068); Post-LP 1: *M* = 0.538 (*SE* = 0.063); Post-LP 2: *M* = 0.645, *SE* = 0.060]. When both congruent and incongruent MEPs are examined, the reduction in MEP size immediately post-training is in direct contrast with our expectations. Indeed, it will be difficult to show AMR across pre- and post-training comparisons if, overall, post-training MEPs are reduced when compared with baseline measurements (see Supplementary Materials for discussion of repetition suppression during the LP blocks, which might explain this unanticipated finding).

Regarding AMR illustration, no Audition block × Sound × Time point interactions were present. This suggests that AMR did not develop. Trained sounds did not increase finger-corticospinal networks beyond baseline measures when all blocks and time points are considered.

However, we were concerned with the use of static TMS trigger points, which do not consider individual variability and learning. We suspected these triggers might be censoring the AMR illustration. If sensorimotor associations are experience dependent, and a participant learns to complete the swipe in 450 ms, then the largest MEPs for the congruent sounds might be generated at this 450 ms time point. Another participant, however, may produce a swipe duration of 300 ms. Thus, the 300 ms time point might be better suited at revealing AMR for this person. Others still, might encode the swipe initiation with the sound, which suggests the 50 ms time point might be suitable. Therefore, examining the MEPs without regard for individual variability could conceal the AMR illustration. Add to this the surprising finding regarding the reduced MEPs immediately post-training, and it perhaps explains why AMR was not revealed.

Accordingly, we examined changes in MEPs across congruent and incongruent sounds for both fingers *via* pre and post-learning blocks separately (for a related examination, see Ticini et al., 2011, 2019). Furthermore, we selected a single time point to overcome the stated challenges with individual variability. We supposed, if (a) individuals learn to associate a finger movement with a sound (e.g., the index finger with the Congruent_FDI_ sound), and (b) behavioral learning variabilities cause changes in the timing of the sensorimotor association process. Perhaps, then, (c) we should explore the inhibition of the incongruent sound MEPs relative to maximum congruent sound MEP at a given TMS time point. In other words, we were interested in the maximal dissociation of congruent vs. incongruent sound-generated MEPs across post-blocks within a time point.

We determined where a participant’s maximal congruent sound MEP in either Post-LP block was recorded. This time point then became the *guide*. We obtained the congruent and incongruent MEPs for both Post-LP blocks at this time point only. A 2 (*Sound*: MAX-congruent and incongruent) × 2 (*Block*: Post-LP 1 and 2) ANOVA for each muscle was run. Demonstration of AMR would show that listening to incongruent sounds generates smaller, perhaps inhibited, MEPs when compared to congruent, trained sounds.

Provided the AMR illustration is time-locked to maximal trained muscle activity during audition, the index finger ANOVA with post-learning blocks demonstrated a significant main effect for Sound (*F*_(1,17)_ = 26.987, *p* < 0.001, $\eta_{\text{p}}^{2}$ = 0.614). PCs (Bonferroni corrected) showed hearing the congruent sound produced larger MEPs (*M* = 1.404, *SE* = 0.143) than the incongruent sound within a time point (*M* = 0.744, *SE* = 0.101). This indicates that AMR develops, and trained sounds generate larger MEPs in corticospinal tracts than those recorded upon hearing untrained (incongruent) sounds.

There was also a significant main effect for Post-LP block (*F*_(1,17)_ = 12.322, *p* = 0.003, $\eta_{\text{p}}^{2}$ = 0.420). PCs (Bonferroni corrected) revealed the Post-LP 1 block generated smaller MEPs (*M* = 0.847, *SE* = 0.125) than those recorded within the Post-LP 2 (*M* = 1.301, *SE* = 0.123). Perhaps this modulation of MEPs across blocks reveals a memory consolidation period, which is facilitated by the TMS pulse and sound playback. Sound playback during TMS to M1 over the FDI region could aid the formed sensorimotor association. In other words, Post-LP block 1 might act like another training block (although see the “Discussion” section for a caveat to this explanation).

The same procedure followed for the little finger. Despite using the higher threshold muscle to generate the MT, ANOVA revealed a significant main effect for Sound (*F*_(1,17)_ = 19.062, *p* < 0.001, $\eta_{\text{p}}^{2}$ = 0.529). Bonferroni corrected PCs indicated the congruent sound produced larger MEPs (*M* = 0.313, *SE* = 0.234) than those recorded when the incongruent sound is played (*M* = 0.234, *SE* = 0.034). This data supports the index finger AMR illustrations, and suggest sensorimotor associations developed after training.

To limit concerns with *post hoc* statistical biases, we used the respective (i.e., individual’s) congruent time point as the guide and explored Pre-LP MEPs, too. For a powerful AMR illustration to exist, we did not expect to find the post-training disassociation between congruent and incongruent sound-derived MEPs to be present in Pre-LP blocks.

**Figure 4:**
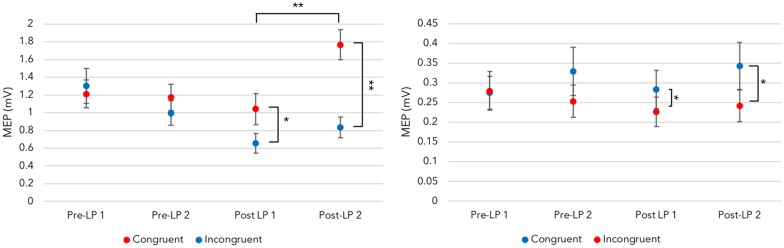
Maximal disassociation of congruent and incongruent sounds across fingers. Left panel: provided the auditory-motor resonance (AMR) illustration is time-locked to the maximal trained motor evoked potential (MEP) time point, a disassociation between congruent index finger MEPs and the incongruent sound-derived MEPs is present after training. This effect, indicating congruent sounds generate larger MEPs than incongruent sounds at an individual time point, is not revealed in the Pre-LP blocks before learning takes place. Right panel: the trained disassociation is also present with MEPs recorded from the little finger [significance levels for *congruency* differences are determined by one-way ANOVAs within a block (see Supplementary Materials for test details); *p < 0.05, **p < 0.001; error bars represent standard error of the mean].

Indeed, separate ANOVAs for each finger do not reveal any main effects or interactions when the Pre-LP MEPs are examined using the guide time point. As shown in Figure 4, a disassociation between congruent and incongruent sound MEPs is only present after learning. This indicates that AMR is a trained effect. Together, this suggests that a bidirectional sensorimotor association developed.

### Testing the Relationship Between Sensory Prediction and Sensorimotor Association

Having established sensory prediction mechanisms during action and AMR during audition, we undertook some nonparametric Spearman correlational analyses to explore how these mechanisms are associated putatively. First, negative correlations between the maximum Post-LP 1 MEPs in the index finger (i.e., audition) and N1 peak data during the little finger-swipe (i.e., action) are present (Fz, *r* = −0.655, *p* = 0.003; FCz, *r* = −0.544, *p* = 0.020). This relationship is mirrored at the Fz electrode with the Post-LP 2 MEPs (*r* = −0.598, *p* = 0.009). Also, the maximum Post-LP 1 MEPs in the index finger and the P2 peak during a little finger-swipe are negatively correlated (Fz, *r* = −0.544, *p* = 0.020; FCz, *r* = −0.610, *p* = 0.007). These data suggest that large index finger MEPs during congruent sound listening are associated with recordings of larger (i.e., less suppressed and more negative) ERP data during the little finger-swipe. Together, this highlights the close ties between a sensorimotor association and sensory prediction during action learning.

In support of this close relationship, behavioral activity and sensory prediction markers also demonstrate some correlation. EMG activity in the index finger during the swipe is negatively correlated with the N1 peak data during audition of the incongruent little finger-sound at Fz (*r* = −0.513, *p* = 0.030) and FCz electrodes (*r* = −0.548, *p* = 0.019). It would seem that efficient index-swipe movements are associated with the absence of early sensory prediction data when hearing the different (related) sound.

Therefore, negative correlations across sensory prediction, sensorimotor association, and behavioral data highlight an interdependent nature of these components. Simply put, the disassociation of closely related motor control plans (e.g., sensorimotor associations) might be achieved *via* sensory prediction processes, which are learnt during behavior.

## Discussion

Few studies have explored how sensorimotor associations develop by way of sensory predictions within a single paradigm. Here, we combined a bespoke device with TMS, EMG, and EEG techniques to explore how these study components were correlated. As expected, our data show increases in P2 and suppression of N2 peaks during action, demonstrating some aspects of sensory prediction outcomes. Also, time-locked AMR disassociations are present, which show that congruent sounds generate larger MEPs than hearing incongruent sounds. These disassociations are considered here to represent precise sensorimotor associations and only appear after learning. Finally, novel findings show that negative correlations between MEPs after learning and ERP data during action are present. Taken together, these results might suggest that sensory prediction mechanisms fine-tune sensorimotor associations, perhaps in line with an internal modeling account of sensorimotor learning.

First, sensory prediction mechanisms are present during action. The N2 peak-component around 200 ms was suppressed during action. Typically, modulation of the N2 is thought to reflect higher-order conflict monitoring (Folstein and Van Petten, 2008), and it is often shown in response to an auditory violation (Kujala et al., 2007). For example, larger N2 components are related to hearing deviant musical notes embedded in known melodies (Mathias et al., 2015, 2016). Here, larger N2 peaks were evident in comparison with auditory-based recordings (i.e., no deviants were heard during sound playback). To reconcile, some suggest the amount of suppression of the N2 peak is contingent upon a comparison between a memory trace and the reafferent information (Näätänen et al., 2007). So, in the context of conflict monitoring or auditory violation, the N2 peak could represent feedback from the reafferent comparison.

Taken further, if the N2 peak represents the amount of *comparative feedback*, does this explain why the earlier P2 peak, approximately 150 ms, was increased during the action? Conceivably, the relatively long action has been able to draw out the sensory prediction outcomes. Perhaps, then, the P2 peak is revealing the comparison or even the prediction itself, rather than some type of signal to maintain the sensory representation during and following suppression (Wang et al., 2014). In doing so, this might explain why the P2 component here was increased. To clarify, sensory predictions should be generated first, before comparison with reafferent sensory stimuli. The comparison should then produce some feedback. Therefore, a sensory prediction mechanism should have three main processes: prediction, comparison, and feedback.

In a practical sense, a swipe movement is made, and an epoch of EEG activity is recorded. Simultaneously, a prediction is made (e.g., expect the index swipe-sound). Meanwhile, motor preparations for the swipe-termination component (e.g., lift-off) are initialized and executed. When available, this prediction or a negative image copy (Ramaswami, 2014; Barron et al., 2017; Enikolopov et al., 2018) is compared with the reafferent auditory stimuli (e.g., a comparison will determine if that was the index swipe-sound). Finally, feedback is provided to motor control areas. We suspect this feedback is in the form of an N2 peak here. If the N2 represents feedback from the predictive comparison, we wonder whether the P2 component might then represent a preceding stage of the prediction process. This could be either the prediction (centrally located) or even the comparison with sensory reafference (perhaps located of auditory-parietal regions).

While we do acknowledge assigning specific processes to individual ERP components is difficult (for a related discussion on complexities of ERP analyses, see Horváth, 2015; Spriggs et al., 2018), we suggest the long action and effect might have been able to tease out some of these separate prediction stages. In contrast, fast actions like a button press (Bäss et al., 2008; Baess et al., 2011; Ford et al., 2014) or short speech sounds (Heinks-Maldonado et al., 2005), might conflate these separate sensory prediction processes into a single N1 outcome. In that case, the time window of action, prediction, and the feedback-response are so small that EEG recordings might not be able to show the underlying computations. In any case, we find evidence for some sensory prediction processes during action.

Sensorimotor associations were also present. As hypothesized, when the learned sounds are heard, activation of related corticospinal circuits that are involved in the associated actions are revealed. This is supported by published data regarding sensorimotor association that indicate training can lead to AMR (Butler et al., 2011; Butler and James, 2013; D’Ausilio et al., 2014; Furukawa et al., 2017).

More specifically, the AMR response was shown *via* a time-locked dissociation between congruent and incongruent sound-derived MEPs. When training generates the largest AMR response for the congruent sound, hearing an incongruent sound generates less activation in the motor circuit. This type of AMR illustration is also supported by other published works on AMR congruency (Ticini et al., 2011, 2019).

Importantly, this type of AMR disassociation is not present before learning. It develops because of sensorimotor experience and appears predicated on behavioral variability; albeit, a *post hoc* and simple delineation of individual differences. That is, the disassociation between congruent and incongruent sounds are not revealed *via* the static TMS time points. Only when an individual time point for the maximally trained (congruent) response is used as an index or guide does AMR appear after learning and not before. Given this, it would seem the sensorimotor association here is more complicated than just a broad cause and effect relationship between motor and sound information. Add to this the sensory prediction data regarding different stages of the predictive process, and we suspect an internal modeling process might be generating the sensorimotor associations.

Indeed, other interpretations can explain how sensory and motor information are associated, such as an Association account (for review e.g., see Cook et al., 2014), Ideomotor perspective (for review e.g., see Herwig, 2015), or even more contemporary prediction theories (Friston, 2010, 2011; Friston et al., 2011, 2017; Pickering and Clark, 2014). However, we focus here on a conventional internal modeling perspective.

Simply put, an internal model mimics the behavior of actions and their consequences within the CNS (for review e.g., see Miall et al., 1993; Miall and Wolpert, 1995; Wolpert et al., 1995, 2011; Wolpert and Kawato, 1998; Wolpert and Ghahramani, 2000; Grush, 2004; Burgess et al., 2017). Traditionally, they consist of an inverse or controller unit that causally integrates sensory consequences with the actions and motor commands that produce them. Second, there is a forward component, which generates predictions regarding upcoming sensory change given the outgoing motor commands. Principally, the combination of inverse and forward components helps overcome temporal delays when integrating sound and action, given large sensorimotor loops (Wolpert et al., 1995; Wolpert and Kawato, 1998). They do this by reducing error in the underlying component mappings through a comparison between predicted and produced sensory stimuli. In turn, feedback updates both model components. In other words, sensory predictions help to overcome the delays in sensorimotor feedback loops when integrating sound and action. Over time, fewer cortical resources are needed to produce an action as the models increase in accuracy and efficiency.

In support of the interdependence of components expected under an internal modeling account, recent studies have shown that sensory predictions are involved with activating sensorimotor association and motor representations (Gordon et al., 2018). Indeed, Stephan et al. (2018) demonstrated that anticipatory MEPs were produced upon hearing sounds in a musical sequence after learning (i.e., sound sequences automatically cued future movement in specific finger muscles). Others have even implicated the cerebellum in hand-reaching experiments when inverse and forward models work in tandem to overcome behavioral adaptation (Honda et al., 2018). Altogether, we suspect the *post hoc* explorations are necessary here to find AMR because sensorimotor associations are very sensitive to individual timing and temporal delays during the action-learning process. In turn, this type of association relies on feedback from the reafferent comparisons *via* sensory prediction, which is shown *via* peak modulation and correlational data across study components. As is, we suspect sensory predictions fine-tune the sensorimotor associations during learning as expected by internal modeling.

Finally, we recognize the complexity of demonstrating these relationships across the sensorimotor divide and concede some methodological issues, which future investigations may wish to consider. Measuring AMR should accommodate individual learning variability. More detailed indices of the behavior (e.g., swipe time and distance) should be recorded and used to inform, for example, TMS-triggering schedules in real time. In turn, more accurate recordings of the AMR time course might mitigate problems with *post hoc* time point selection. Also, more accurate TMS triggers might help explain the decrease in Post-LP 1 MEPs, recorded immediately after training. Some TMS studies have indicated that repetitive finger movements can decrease MEPs when measured immediately after training (i.e., 1–2 min), even without fatigue (McDonnell and Ridding, 2006; Avanzino et al., 2011; Kluger et al., 2012; Miyaguchi et al., 2017). While we applied a 4-min break between training and TMS assessment during listening, not to mention the obligatory 3 s break between swipes, this might not have been long enough to overcome the supposed *post-exercise depression* in MEPs when using static time points.

Additionally, change in the movement should also trigger EEG equipment such that the protocol can determine when an *error* occurred. For instance, a movement that was outside of a typical participant response may help clarify more precisely how behavior affects sensory prediction, which in turn affects AMR development. Alternatively, it might show how these prediction processes develop during learning (e.g., what happens to ERP peak modulation as behavior goes from atypical-to-typical movements). Similarly, future sensory prediction studies might wish to examine motor-related potentials more closely, which do not subtract the motor trace from the ERP. In doing so, understanding the brain dynamics of sensory prediction during action might be more achievable. Future studies might also wish to consider if differences in stimulus sequences and inter-stimulus intervals across action and audition stages affect the ERP traces. Indeed, there are many questions that remain when analyzing sensory prediction with EEG (Horváth, 2015). Finally, there is evidence to suggest that the menstrual cycle may affect motor cortex excitability when measured *via* TMS (Hattemer et al., 2007; Pellegrini et al., 2018). Thus, future studies should take this into account.

The problem with attempting to measure these internal modeling mechanisms in humans goes beyond the simple inferences of *gross* or system-level recordings. Instead, methodological protocols and technology that can measure, record, and reflect those processes as they develop should be used. As is, we can only assume that our data measure these modeling processes rather than something more straightforward.

## Summary

Overall, we have documented putative sensorimotor association or AMR development from a sensory prediction and internal modeling perspective. In the present study, sensory prediction indices are present in the form of enhanced P2 and suppressed N2 peaks during action. These might represent different stages of the prediction process. Also, novel sensorimotor associations develop and appear tuned. Hearing congruent sounds generates larger MEPs than those recorded during incongruent sound listening once time-locked and within a TMS time point. Importantly, these disassociations are not present before learning, suggesting that AMR and sensorimotor associations are experience dependent. Finally, there appears to be a relationship between the strength of a sensorimotor association measured during listening and how a related, yet incongruent, sound is predicted during action. In other words, sensory predictions seem to affect how precise a sensorimotor action is encoded. While future investigations may wish to examine behavioral indices further, we consider our data to represent a preliminary step towards understanding how, and perhaps why, sensory signals activate motor brain regions.

## Ethics Statement

All procedures performed in studies involving human participants were in accordance with the ethical standards of the institutional and national research committee, and with the 1964 Helsinki declaration and its later amendments or comparable ethical standards. The research was approved by Deakin University Human Research Ethics Committee (2015-034).

## Author Contributions

JB and PE: experiment design. JB, BM, and CM: data collection. JB: data analysis. JB, GC, JL, and PE: wrote the article.

## Conflict of Interest Statement

The authors declare that the research was conducted in the absence of any commercial or financial relationships that could be construed as a potential conflict of interest.
