# Supplementary material for: Learning to Expect: Predicting Sounds During Movement Is Related to Sensorimotor Association During Listening
Source: Front Hum Neurosci. 2019 Jul 4;13:215. doi: 10.3389/fnhum.2019.00215 (PMC6624421; doi:10.3389/fnhum.2019.00215)
Supplement: Supplementary file 1 [file Table_1.pdf]

Supplementary Materials

Hypothesis testing

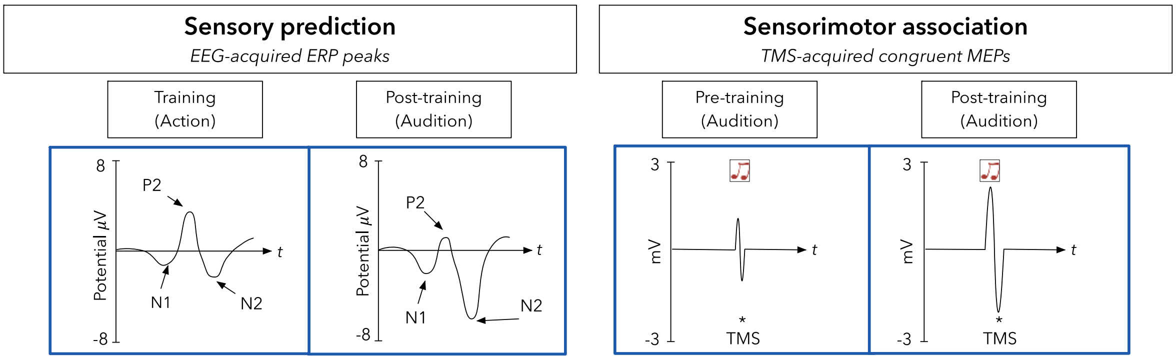

Figure S1. *Hypothesis testing. Left panel*: Firstly, we sought to examine sensory prediction markers via electroencephalography. We hypothesise smaller minimum N1 or N2 event-related potential (ERP) peaks will be recorded during a swipe (far left box) compared to audition post-training (centre-left box). We also hypothesise larger positive P2 peaks will be produced during the action. *Right panel*: Next, we hypothesise that sensorimotor associations, revealed via transcranial magnetic stimulation, will generate larger motor evoked potentials from finger muscles upon hearing the congruent sounds after training (far right box). Finally, we hypothesise that some negative correlations between ERP data, EMG, and MEPs will be present.

Swipe movements

To potentially minimise the problems with prior learning, which might be associated with button presses or more everyday-finger actions, we opted to use swipe movements. These movements were shown in planning to facilitate large activations of the index and finger muscles, which were considered crucial for measuring the potential sensorimotor associations.

While the swipe movements were designed to be novel and activate each muscle sufficiently, we concede the actions are complicated enough to contain potential inherent variability, both within and across participants.

Static TMS latencies and potential caveats

Typically, AMR is determined in *a priori* manner before learning has taken place. Perhaps owing to technological constraints, TMS time points are often calculated before an experiment begins. As a result, these presupposed and instantaneous recordings may not measure individual AMR temporal properties. Instead, these TMS time points are static and do not consider behavioural outcomes, which might change after training. By avoiding the dynamic nature of sensorimotor learning, it could be argued that these time points assess more general learning principles. Nevertheless, no studies to our knowledge have provided some suggestion of possible solutions to overcome the use of static TMS time points when assessing sensorimotor associations.

Sounds

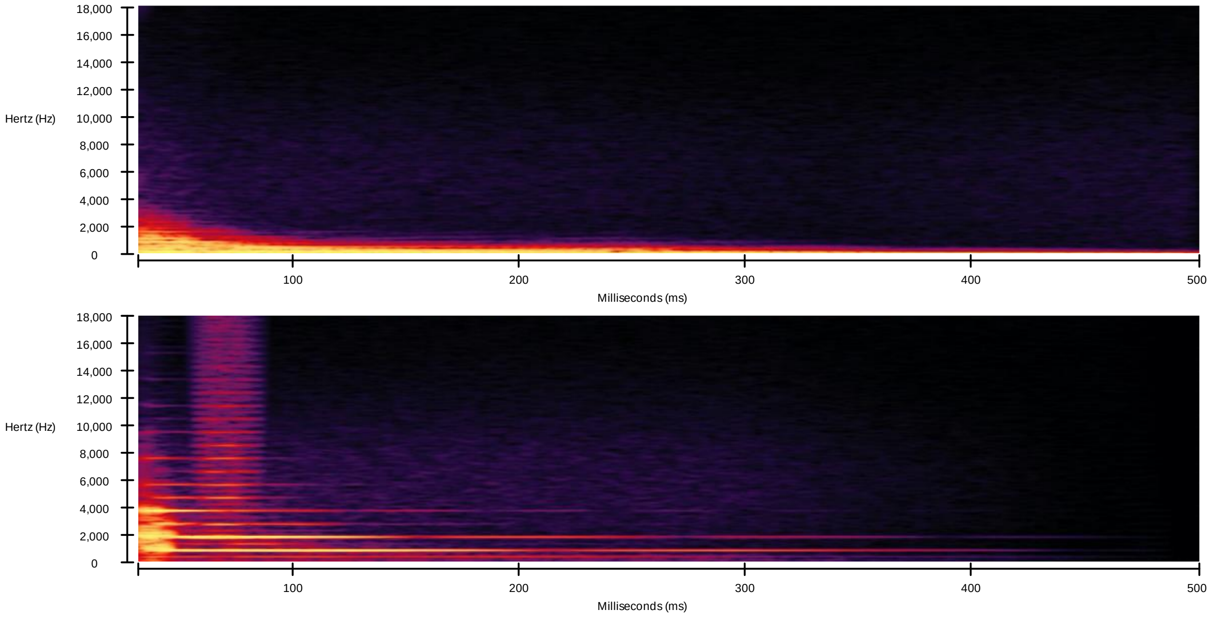

Figure S2. *Sound spectrograms over time*. *Top panel:* The *low* sound consisted of an approximate 500 Hz fundamental tone, while 250 Hz and 1000 Hz overtones were present. *Bottom panel:* The *high* tone consisted of an approximate 1250 Hz fundamental tone, as well as 2100 Hz and 4000 Hz overtones. Sounds were 500 ms in duration and played via in-ear headphones.

The AMRJ and trigger design

To indicate sound playback on recording devices following switch activation in the Action stage, 5V TTL pulses were sent to trigger EMG recordings via 1 (of 2) x BNC connectors (i.e., TMS was not used during the action stage). Simultaneously, a similar 5V TTL pulse was transmitted via a 25-pin D-type serial cable and specific stimulus pins as a trigger for EEG recordings (see Figure S3 below for an illustration of the trigger design; see the main paper for more details regarding TMS, EMG, and EEG recordings).

During the Audition stage, quasi-randomised sequences of 48 sound samples were played via the in-ear headphones. At the same time, 5V TTL pulses were sent to trigger EMG and TMS equipment via 2 x BNC connectors, while a similar 5V TTL pulse was sent via the serial cable to trigger EEG recordings.

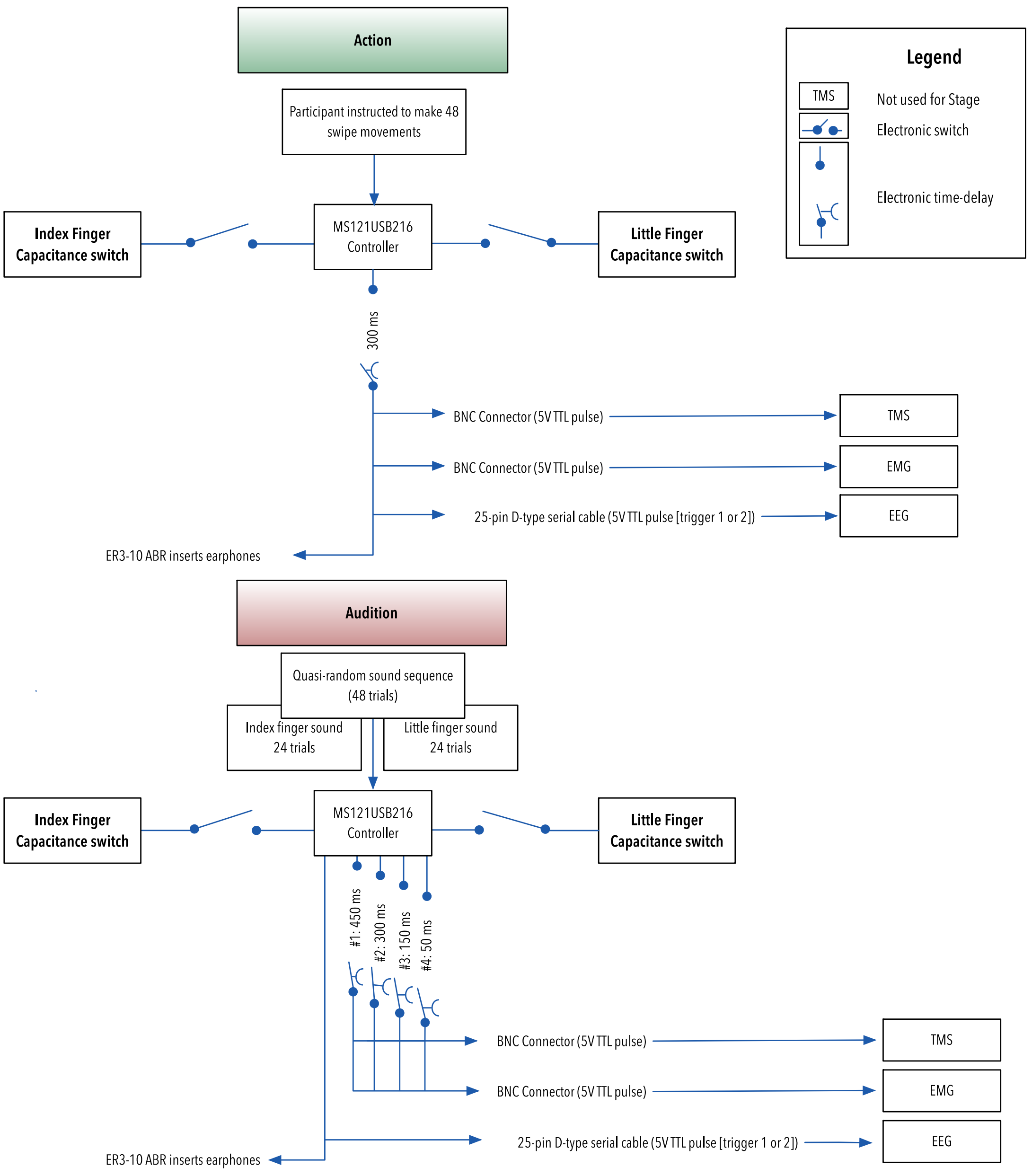

Figure S3. *Trigger-circuit design for the AMRJ.* Illustrated here is the trigger-circuit design for the AMRJ across both experimental stages.

Extended data tests

Repetition suppression during training and potential influence of MEPs Post-LP 1

We designed the AMRJ to trigger EMG recordings during finger swipes. This capacity was added to the device architecture to gain insight into repetition suppression (RS) profiles during learning. Indeed, the reduction in cortical response following repeated presentation of sensory stimuli is well demonstrated (for review example see, Grill-Spector et al., 2006). Some studies have even shown that repeated sensorimotor action can generate similar

reductions in cortical and corticospinal activity to those seen during frequent sensory observation (Dinstein et al., 2007; Hamilton and Grafton, 2009).

We suspected if (a) AMR developed after the LP blocks and (b) changes in sensory prediction mechanisms are observed during the LP blocks, then (c) we could see some modulation of EMG activity (indexed as a reduction in corticospinal excitability) across the LP blocks (e.g., start-to-finish). We used capacitance switches, which do not require force to activate, also for this reason. We expected the lack of a force-hurdle during switch activation might help illustrate this RS paradigm (i.e., a minimum force was not required to the press a switch; therefore, assessing the reduction in CSE during learning was very sensitive using this type of paradigm). In short, we sought to explore whether the CNS became more efficient at producing the same sensory outcome near the end of the training.

To explore if RS was present during the LP block recordings, we obtained EMG recordings from the index and little-finger muscles during each respective swipe. A mean value was generated from all the *active* swipes within each LP block (e.g., an index and a little-finger mean EMG value for LP 1-4). To compare, we ran a 2 (Finger: Index and little) x 4 (Block: LP 1, 2, 3, and 4) ANOVA.

Figure S4 depicts the mean (peak-to-peak) EMG data (mV) recorded at SOA from each muscle during the congruent swipe (e.g., *active* swipe) across blocks. Demonstrating a reduction in overall motor system excitability to achieve the same sensory outcome, ANOVA revealed a significant main effect for *LP block* ($F_{3, 51} = 9.401$, $p < .001$, $\eta_p^2 = .356$). PCs, which were Bonferroni corrected, indicated that EMG activity generated during LP 1 were significantly larger than LP 3 ($p = .007$) and LP 4 ($p = .009$), while LP 2 was significantly larger than LP 4 ($p = .046$). Also, LP 2 was approaching significance when compared to the smaller LP 3 data ($p = .051$). As expected, no differences between muscles were shown ($F_{1, 17} = .015$, $p = .903$, $\eta_p^2 = .001$), nor were any interaction terms significant ($F_{3, 51} = .395$, $p = .757$, $\eta_p^2 = .023$). Together, these data suggest that RS was present during the learning process.

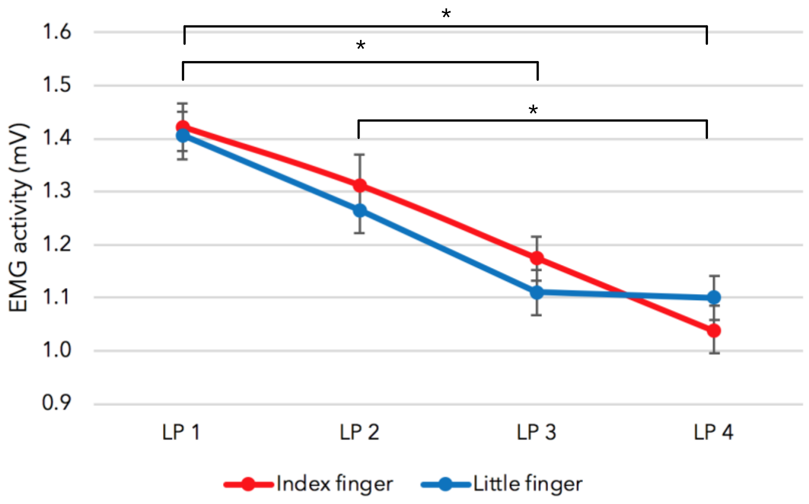

Figure S4. *Modulation of EMG activity during LP blocks.* During swipe movements,

electromyography (EMG) recordings were taken to explore a repetition suppression-like influence or the reduction in CNS activity following repeated execution. ANOVA revealed Learning Phase (LP) 1 data were significantly larger than LP 3 and LP 4 EMG activity, while LP 2 produced larger recordings than LP 4. Since data demonstrates behavioural habituation following repeated sensorimotor experience, these results might illustrate repetition suppression effects via predictive mechanism development (* denotes $p < .05$; error bars represent standard error of the mean).

Discussion of repetition suppression during training and potential influence of MEPs Post-LP 1

The reduction in cortical response following repeated presentation of sensory stimuli is known as repetition suppression (RS). Some accounts of RS suggest this reduction during processing might be a result of neural fatigue and the depletion of resources associated with neuronal firing (Kuravi and Vogels, 2017). Indeed, this could explain why EMG activity reduced and perhaps even why overall MEPs immediately after training were smaller in Post-LP 1. That is, the neural circuits responding to the sounds during action training became depleted in the late stages of learning; thus, the CNS responded poorly when subsequently processing the sounds. However, breaks between blocks were intended to reduce the impact of fatigue on cortical and corticospinal networks. Furthermore, Post-LP 2 MEPs returned, which suggest that neural fatigue of associated circuits was not an issue.

An alternative explanation for the reduction in EMG activity during learning might involve neural sharpening (Gotts et al., 2012) or even inhibitory sharpening (Spigler and Wilson, 2017). According to these perspectives, poorly tuned neurons are disassociated or removed via repeated stimuli presentation. Subsequently, this process reduces the activation of networks during sensorimotor and sensory processing.

Here, EMG activity during the active swipe is reduced throughout training. This suggests that corticospinal networks required less *activity* to produce the same sensory outcome and generate the sounds. In other words, inefficient neural connections were discarded during training, and there is a subsequent increase in the precision of the corticospinal network. This increase in precision is demonstrated as the time-locked AMR between trained and untrained sounds. Indeed, this accuracy in AMR recordings (between congruent and incongruent sounds) is not present before learning has taken place. Together, the reduction in corticospinal excitability during learning and the increases in AMR congruency after learning suggest inefficient neural pathways are removed and discarded.

More recently, some have even suggested that RS processes might be underpinned by changes in sensory prediction mechanisms (for review examples see, Friston, 2010; Clark, 2013; Auksztulewicz and Friston, 2016; Trapp and Kotz, 2016). As noted (see main text), sensory prediction mechanisms are typically thought to involve a reafferent comparison. Firstly, a sensory prediction is generated according to the outgoing motor plans (e.g., it is predicated on the sensorimotor association, which generates movement plans). In turn, this prediction is compared with reafferent stimuli, and feedback is produced. Over time, feedback updates the associations and predictions to ensure the action is accurate and

efficient. In other words, as the predictions become more accurate, less feedback should be produced during the reafferent comparison. Thus, less feedback could represent less cortical activity (e.g., RS) because sensory information is *explained away* more accurately once networks reduce prediction error (Clark, 2013).

Taken together, we suspect the increased suppression of the N2 peak during action is indicative of a reduction in prediction error. Also, we suggest this reduction in error is related to a decrease in EMG activity in the late stages of learning. In turn, inefficient neural pathways during the finger-swipe movement are discarded, and an increase in precision of the network is shown post-learning as the developed disassociation between MEPs.

Testing TMS' influence on CSE

Finally, there are reports of an initial or early transient-state influences of single-pulse TMS on corticospinal excitability (Schmidt et al., 2009). Also, some data have shown that single-pulse TMS has cumulative effects on MEP recordings (Pellicciari et al., 2016). Therefore, we ran a one-way ANOVA assessing baseline MEPs across the three blocks with the TMS-experimental control stage. ANOVA revealed no main effect ($F_{1.33, 22.52}$ = 0.684, p = 0.457, η_p^2 = 0.039; Greenhouse-Geisser corrected ε = .662), indicating no transient state or cumulative differences in baseline corticospinal excitability across the experiment were discovered.

Table 1. Tests details

Columns indicate the results *source*, *df*, *F* value, *p*-value, *partial Eta Squared* value, and the *Greenhouse-Geisser* statistic. Bold text denotes a significant finding. Where main effects are present, mean (*M*) and standard error (*SE*) of the mean data are provided.

Source	df	F	Significance	Partial Eta Squared	Greenhouse-Geisser
**Testing sensory prediction**					
**N1 ERP suppression across action and audition stages via Fz and FCz electrodes ([2] Finger x [2] Stage x [2] Electrode)**					
Finger	1,17	4.216	.056	.199	
Stage	1,17	.836	.373	.047	
Electrode	1,17	.035	.853	.002	
Finger x Stage	1,17	.043	.838	.003	
Finger x Electrode	1,17	.057	.814	.003	
Stage x Electrode	1,17	.937	.347	.052	
**Finger x Stage x Electrode***	**1,17**	**6.031**	**.025**	**.262**	
*Multiple ANOVAs were run to investigate this 3-way interaction. ANOVA demonstrated another 3-way interaction for the little finger ($F_{1, 17}$ = 7.198, *p* = .016, η_p^2 = .297). However, further exploration did not reveal the source of the interaction, suggesting the difference was limited.					
**P2 ERP suppression across action and audition stages via Fz and FCz electrodes ([2] Finger x [2] Stage x [2] Electrode)**					
Finger	1,17	1.662	.215	.089	
Stage	1,17	4.471	.050	.208	
PC—Action > Audition					
Action: *M* = 2.704, *SE* = .653					
Audition: *M* = 1.438, *SE* = .486					
Electrode	1,17	8.271	.010	.327	
PC—FCz > Fz					
FCz: *M* = 2.218, *SE* = .504					
Fz: *M* = 1.924, *SE* = .484					
Finger x Stage	1,17	.102	.753	.006	
Finger x Electrode	1,17	.631	.438	.036	
Stage x Electrode	1,17	.425	.523	.024	
Finger x Stage x Electrode	1,17	.219	.646	.013	
**N2 ERP suppression across action and audition stages via Fz and FCz electrodes ([2] Finger x [2] Stage x [2] Electrode)**					
(N2) Finger	1,17	.212	.651	.012	
**Stage**	**1,17**	**15.296**	**.001**	**.474**	
**PC—Audition > Action**					
Audition: *M* = -9.359, *SE* = .738					
Action: *M* = -6.676, *SE* = .435					
**Electrode**	**1,17**	**22.161**	**< .001**	**.566**	
**PC—FCz > Fz**					
FCz: *M* = -8.518, *SE* = .561					
Fz: *M* = -7.516, *SE* = .456					

Finger x Stage	1,17	.249	.624	.014	
Finger x Electrode	1,17	.181	.676	.011	
**Stage x Electrode***	**1,17**	**17.112**	**.001**	**.502**	
Finger x Stage x Electrode	1,17	.080	.781	.005	

*Further exploration was not conducted as more tests were deemed outside of the paradigm scope.

Testing sensorimotor associations

AMR via time point and sound comparisons from before and after training blocks ([2] Finger x [4] Audition block x [4] Timepoint x [2] Sound)

**Finger**	**1, 17**	**82.662**	**< .001**	**.829**	
**PC—Index > Little**					
Index: M = 1.043, SE = .096					
Little: M = .255, SE = .032					
**Audition block**	**3, 51**	**3.799**	**.016**	**.183**	
**PCs—Did not survive Bonferroni adjustments**					
Estimated marginal means					
Pre-LP 1: M = .708, SE = .076					
Pre-LP 2: M = .704, SE = .068					
Post-LP 1: M = .538, SE = .063					
Post-LP 2: M = .645, SE = .060					
**Timepoint**	**3, 51**	**4.575**	**.007**	**.212**	
**PC—50 ms > 150 ms**			**.003**		
50 ms: M = .681, SE = .060					
150 ms: M = .612, SE = .051					
Sound	1, 17	.493	.492	.028	
Finger x Audition block	3, 51	2.767	.051	.140	
**Finger x Timepoint***	**3, 51**	**4.055**	**.012**	**.193**	
Audition block x Timepoint	9, 153	1.348	.217	.073	
Finger x Sound	1, 17	.153	.701	.009	
Audition block x Sound	3, 51	1.742	.170	.093	
Timepoint x Sound	3, 51	.630	.599	.036	
Finger x Audition block x Sound	3, 51	.179	.910	.010	
Finger x Audition block x Timepoint	9, 153	.818	.600	.046	
Finger x Timepoint x Sound	3, 51	.372	.773	.021	
Audition block x Timepoint x Sound	4.639, 78.863	.550	.725	.031	.515
Finger x Audition block x Timepoint x Sound	9, 153	.433	.915	.025	

*Further exploration was not conducted as more tests were deemed outside of the paradigm scope.

Maximal dissociation between congruent and incongruent sounds in Post-LP blocks—separated by Finger ([2] Sound x [2] Post-LP blocks)

FDI

**Sound**	**1, 17**	**26.987**	**< .001**	**.614**	
**PC—Congruent (max) > Incongruent within the timepoint**					

Congruent: *M* = 1.404, *SE* = .143				
Incongruent: *M* = .744, *SE* = .101				
**Post-LP block**	**1, 17**	**12.322**	**.003**	**.420**
**PC—Post-LP 2 > Post-LP-1**				
Post-LP 2: *M* = .847, *SE* = .125				
Post-LP 1: *M* = 1.301, *SE* = .123				
**Sound x Post-LP block***	**1, 17**	**9.653**	**.006**	**.362**
*Further examination of this two-way interaction was not conducted owing to the main effect for Sound.				
**Maximal dissociation between congruent and incongruent sounds in Post-LP blocks —separated by Finger ([2] Sound x [2] Post-LP blocks)**				
**ADM**				
**Sound**	**1, 17**	**19.062**	**< .001**	**.529**
**PC—Congruent (max) > Incongruent within the timepoint**				
Congruent: *M* = .313, *SE* = .046				
Incongruent: *M* = .234, *SE* = .034				
Post-LP block	1, 17	.708	.412	.040
Sound x Post-LP block	1, 17	1.937	.182	.102
**Maximal dissociation between congruent and incongruent sounds using the Post-LP timepoint within the Pre-LP blocks—separated by Finger ([2] Sound x [2] Pre-LP blocks)**				
**FDI**				
Sound	1, 17	.103	.753	.006
Pre-LP block	1, 17	2.033	.172	.107
Sound x Pre-LP block	1, 17	2.253	.152	.117
**Maximal dissociation between congruent and incongruent sounds using the Post-LP timepoint within the Pre-LP blocks—separated by Finger ([2] Sound x [2] Pre-LP blocks)**				
**ADM**				
Sound	1, 17	1.896	.186	.100
Pre-LP block	1, 17	.125	.728	.007
Sound x Pre-LP block	1, 17	2.975	.103	.149
**Supplementary Materials**				
***One-way ANOVAs for Figure 4 and determining significance values of congruence within each block**				
**Post-LP 1 & Index finger: ([2] Sound: MAX-congruent and incongruent)**				
**FDI**				
**Sound**	**1, 17**	**6.178**	**.024**	**.267**
**PC—Congruent (max) > Incongruent within the timepoint**				
Congruent: *M* = 1.041, *SE* = .177				
Incongruent: *M* = .654, *SE* = .111				
**Post-LP 2 & Index finger: ([2] Sound: MAX-congruent and incongruent)**				
**FDI**				
**Sound**	**1, 17**	**37.122**	**< .001**	**.686**
**PC—Congruent (max) > Incongruent within the timepoint**				
Congruent: *M* = 1.767, *SE* = .168				

	Incongruent: *M* = .835, *SE* = .117					
**Post-LP 1 & Little finger: ([2] Sound: MAX-congruent and incongruent)**						
**ADM**						
**Sound**		**1, 17**	**11.062**	**.004**	**.394**	
	**PC—Congruent (max) > Incongruent within the timepoint**					
	Congruent: *M* = .283, *SE* = .048					
	Incongruent: *M* = .226, *SE* = .037					
**Post-LP 2 & Little finger: ([2] Sound: MAX-congruent and incongruent)**						
**ADM**						
**Sound**		**1, 17**	**11.965**	**.003**	**.413**	
	**PC—Congruent (max) > Incongruent within the timepoint**					
	Congruent: *M* = .343, *SE* =.059					
	Incongruent: *M* = .242, *SE* = .040					
**Potential repetition suppression influence on EMG activity during LP blocks: ([2] Finger x [4] LP blocks)**						
Finger		1, 17	.015	.903	001	
**LP block**		**3, 51**	**9.401**	**< .001**	**.356**	
	**PC—LP 1 > LP 3**			**.007**		
	LP 1: *M* = 1.414, *SE* = .146					
	LP 3: *M* = 1.142, *SE* = .121					
	**LP 1 > LP 4**			**.009**		
	LP 1: *M* = 1.414, *SE* = .146					
	LP 4: *M* = 1.069, *SE* = .097					
	**LP 2 > LP 4**			**.046**		
	LP 2: *M* = 1.289, *SE* = .130					
	LP 4: *M* = 1.069, *SE* = .097					
Finger x LP block		3, 51	.395	.757	.023	
**Potential influence of TMS on CSE in Baseline blocks ([3] Baseline blocks)**						
Baseline block		2, 34	.684	.457	.039	.662
	B 1: *M* = 1.230, *SE* = .526					
	B 2: *M* = 1.404, *SE* = .616					
	B 3: *M* = 1.283, *SE* = .536					
